# Data on information sources, knowledge and practice on hepatitis B virus in southwest Nigeria

**DOI:** 10.1016/j.dib.2020.105507

**Published:** 2020-04-09

**Authors:** Evaristus Adesina, Olusola Oyero, Nelson Okorie, Lanre Amodu, Babatunde Adeyeye, Darlynton Yartey

**Affiliations:** Department of Mass Communication, Covenant University, Nigeria

**Keywords:** Hepatitis B, Health communication, Information use, Nigeria

## Abstract

In response to the global call for strategic information to understand viral hepatitis, the dataset provides information on the use information sources on hepatitis B virus (HBV) by residents of Southwest Nigeria. The data further shows the knowledge and practice level of residents on HBV. The data were generated among 582 respondents residing in suburban region of Southwest of Lagos, Ogun and Oyo states through a self-administered questionnaire. The data found out that residents of Southwest Nigeria obtained information on Hepatitis B predominantly from the internet (mean score: 3.0687and std. dev. 1.3604). Furthermore, residents of Southwest Nigeria had sufficient knowledge on hepatitis B infection (mean score of 3.239; std. dev. 0.7481). In addition, most of the respondents depicted a positive secondary preventive practice such as insistence on the use of sterilised object in body piercing, demanding for change of blade, needle from hairstylist, screening before blood transfusion (mean score 2.9874; std. dev. 0.7488). The data utilized the Statistical package for social sciences (SPSS) coding the data. Cronbach Alpha was used in carrying out the reliability of the research instrument. Descriptive analysis was further employed in data presentation.

Specifications table

**Table:** 

Subject area	*Media Technology*
More specific subject area	*Health Communication*
Type of data	*Primary data (Tables)*
How data was acquired	*Survey through the use of a questionnaire*
Data format	*Raw, analyzed, descriptive research design*
Parameters for data collection	*The data for the survey were obtained from respondents in the three state with the most prevalent cases of hepatitis B in Southwest Nigeria.*
Description of data collection	*Data was collected through primary survey. Questionnaire is attached to the article.*
Data source location	*Region: Southwest* *Country: Nigeria*
Data accessibility	*Dataset is included with this article*

## Value of the data

1. The data can serve as a basis for further research on health communication and hepatitis management
2. The data can improve the behavioural practice of test and vaccination against hepatitis B virus.
3. The analyzed data can provide suggestions on ending endemic diseases of hepatitis by the year 2030 as contained in the SDG 3.
4. The data shared provides a focal direction for heath communication experts, researchers and policy makers in directing their campaigns for effectiveness.

## 1 Data description

Globally, viral hepatitis disease is a highly endemic public health problem, similar to other communicable diseases, including HIV, tuberculosis and malaria. The World Health Organization has estimated about 257 million people living with chronic HBV [1]. In the African region all hepatitis viruses have been noted to be in existent, however hepatitis B is more endemic with its prevalence among 5–8% of the population [2]. The Sub-Saharan Africa has also been reported to have the highest prevalence rate of 6.1% [3]. The 22.6 million seroprevalence level of hepatitis disease in Nigeria, as observed by the Federal Ministry of Health, has been proven by scientific studies conducted over 40 years ago till date [4], [5], [6].

Health communication has been noted to be an effective panacea to not only getting people knowledgeable about endemic diseases, but also forming a positive attitudinal and behavioural practices towards such [7], [8], [9], [10], [11], [12]. The use of communication channels is crucial to engendering optimal use of health information made available by the health instructors as well as improving knowledge and behavioural change. This allows individuals and communities align with healthy living practices and seek proper medical help. The dataset Table 1–3 is on how the residents of Southwest utilised information sources such as mass media channels, interpersonal networks and the internet for hepatitis B; their knowledge as well as the practice towards HBV. The detailed assessment of responses on the use of information sources; knowledge and practice on Hepatitis B by residents of South West Nigeria is depicted in Table 1–3 and tested using the five Likert scale. This helped in determining the respondents’ views and the extent to which they agreed to the items presented in the questionnaire. The Likert scale is represented as: Strongly Agree (SA), Agree (A), Undecided (U), Disagree (D), and Strongly Disagree (SD).

**Table 1:** Descriptive statistics of information sources on Hepatitis B by residents of South West Nigeria.

Selected Variables (Information sources)	N	SA [5] (%)	A [4] (%)	U [3] (%)	D [2] (%)	SD [1] (%)	Mean	SD
Radio provision of information on HBV	582	8.1	29.4	14.4	28.4	19.8	2.7766	1.2821
Television provision of information on HBV	582	8.8	29.7	14.1	27.8	19.6	2.8024	1.2936
Newspaper provision of information on HBV	582	8.8	23.9	17.7	30.2	19.4	2.7234	1.2628
Leaflets/pamphlet /brochure/flyer/catalog provision of information on HBV	582	8.1	18.7	18.6	30.6	24.1	2.5619	1.2606
Internet/ provision of information on HBV	582	16.2	30.1	15.8	20.4	17.5	3.0687	1.3604
Health worker(s) provision of information on HBV	582	15.8	30.4	15.3	21.3	17.2	3.0636	1.3549
Colleague provision of information on HBV	582	8.1	27	16.8	27	21.1	2.7388	1.2816
Neighbor(s) provision of information on HBV	580	4.6	20.1	19.9	32	25.4	2.4656	1.2001
Counsellor provision of information on HBV	582	5.5	18.9	16.3	34.7	24.6	2.4605	1.2043
Relative provision of information on HBV	582	9.3	19.4	17.0	33.3	21.1	2.6254	1.2668
Seminar/workshop/conference provision of information on HBV	582	9.8	21.1	16.2	30.2	22.7	2.6512	1.3015

**Table 2:** Descriptive statistics on knowledge of HBV.

Selected Variables (Knowledge of HBV)	N	SA [5] (%)	A [4] (%)	U [3] (%)	D [2] (%)	SD [1] (%)	Mean	SD
HBV is caused by a virus	582	18.7	35.2	20.3	12.5	13.2	3.3368	1.2827
HBV affect proper functioning of the liver	582	27.8	33	20.4	9.8	8.9	3.6100	1.2366
HBV can be transmittable from mother to foetus	582	14.8	27.5	31.4	15.5	10.8	3.1993	1.1901
HBV can be transmitted through infected sharp objects	582	14.3	26.8	25.6	17.7	15.6	3.0636	1.2805
HBV cause liver cancer	582	16.3	33.2	29.2	11.0	10.3	3.3419	1.1799
Jaundice is a symptoms of HBV	582	11.9	23.7	37.6	14.9	11.9	3.0876	1.1531
HBV transmittable by contaminated water/food prepared by infected person	582	11.0	23.5	29.0	19.2	17.2	2.9192	1.2454
A person can be infected with HIV and HBV at the same time	582	13.6	25.4	33.2	14.9	12.9	3.1186	1.2044
A person can be infected with HBV and not have the physical symptoms of the disease	582	12.4	27.3	29.2	16.3	14.8	3.0619	1.2333
HBV can be spread by having sexual intercourse with an infected person	582	17.5	22.5	26.5	18.9	14.6	3.0945	1.3013
There is a vaccine for HBV	582	20.4	34.7	23.2	10.3	11.3	3.4261	1.2421
HBV can be cured	582	19.2)	35.6	25.8	9.8	9.6	3.4502	1.1866

**Table 3:** Descriptive statistics on the practice of HBV by the respondents.

Selected Variables (Practice on HBV)	N	SA [5] (%)	A [4] (%)	U [3] (%)	D [2] (%)	SD [1] (%)	Mean	SD
I have been screened for hepatitis B before	582	7.6	14.4	16.3	27.8	33.8	2.3402	1.2832
I have been vaccinated against hepatitis B	582	8.1	14.1	18.7	30.2	28.9	2.4227	1.2611
I ask for a new syringe at health facilities if you need to use one	582	17.4	31.1	17.4	19.2	14.9	3.1667	1.3307
I often ask my hair stylist to change blade, needle or sterilize the clipper	582	25.4	32.5	16.3	13.1	12.7	3.4485	1.3353
I often insist on the use of sterilized or safe objects for any form of body piercing (e.g. ear, nose)	582	23.9	38.0	15.6	10.7	11.9	3.5137	1.2864
I ask about screening of blood before transfusion	582	24.4	33.5	17.2	14.1	10.8	3.4656	1.2927
If I am diagnosed with Hepatitis B, I would you proceed on further medical consultations	582	28.2	31.4	16.3	12.9	11.2	3.5258	1.3205
If I am diagnosed with Hepatitis B, I would share food utensils with someone else	582	7.7	16.8	29.9	24.9	20.6	2.6615	1.1998
If I am diagnosed with Hepatitis B, I would avoid meeting with people	582	5.8	12.0	22.0	30.8	29.4	2.3419	1.1858

## 2 Experimental design, materials, and methods

The data was generated from a survey conducted among 582 residents of three Southwest states of Lagos, Oyo and Ogun with the highest prevalence rate of hepatitis B. Umego, et al. [13], while citing Mbaawuaga, et al. [14], revealed that Lagos had a 14% incidence rate of hepatitis B. In Ogun and Oyo states, Anaedobe, et al. [6] put the prevalence rate at 8.0% and 8.3% respectively. Multistage sampling technique was used in delimiting the population to a manageable size. At the first stage, the researcher stratified Oyo, Ogun and Lagos states into senatorial districts. Oyo State has three senatorial districts namely Oyo South, Oyo Central and Oyo North. Ogun East, Ogun Central and Ogun West make up the senatorial districts of Ogun State. While Lagos State has Lagos Central, Lagos West and Lagos East. Using the lottery method of random sampling, the researcher selected two (2) senatorial districts from each state making a total of six (6) senatorial districts. The second stage, the researcher stratified these senatorial districts into local government areas. Two local government areas from each senatorial district were selected. Twelve (12) local governments were selected in all across the states. At the third stage, the researcher further stratified the local governments into wards. Using the lottery method of the simple random sampling technique, two wards were selected from each local government. This makes the total wards selected to be twenty-four (24). Furthermore, the researcher stratified the selected wards into two (2) streets each using the lottery method. This resulted in forty-eight (48) selected streets. In the fifth stage, the streets were stratified into residential houses; the researcher made use of the systematic sampling technique to select the residential houses that fall into the sample. The questionnaire utilized for the data collection was primarily designed by the researchers and distributed in line with what is already established in literature precisely to elicit response on the variables under consideration. Descriptive statistical data, expressed in frequency, percentages and cross-tabulations were utilised in describing the use of information sources; knowledge and practice residents of Southwest Nigeria on hepatitis B. Statistical Package for Social Science (SPSS) 23 was used to analyze the data.

## Appendix Supplementary materials

Image, application 1

Image, application 2

Image, application 3

Image, application 4
