# Supplementary material for: Data on information sources, knowledge and practice on hepatitis B virus in southwest Nigeria
Source: Data Brief. 2020 Apr 9;30:105507. doi: 10.1016/j.dib.2020.105507 (PMC7168725; doi:10.1016/j.dib.2020.105507)
Supplement: Supplementary file 3 [file mmc3.pdf]

Covenant University
Canaanland, Km 10, Idiroko Road, Ota, Ogun State, Nigeria.
E-mail:cucrid@covenantuniversity.edu.ng
Website: www.covenantuniversity.edu.ng
Tel:+234-09033550046

COVENANT UNIVERSITY
Raising A New Generation Of Leaders
THE WORLD UNIVERSITY RANKINGS 2020 TOP 500

OFFICE OF THE DIRECTOR, COVENANT UNIVERSITY CENTRE FOR RESEARCH, INNOVATION AND DISCOVERY
Professor E.E.J. Iweala

13th January, 2020

Data in Brief,

3251 Riverport,

Maryland Heights,

MO 63043

USA.

LETTER OF INTRODUCTION

The article titled **Data on Information Sources, Knowledge and Practice on Hepatitis B Virus in Southwest Nigeria** submitted by ***Evaristus ADESINA et al*** for publication in your reputable journal has gone through the Ethical Committee of Covenant University Centre for Research, Innovation and Discovery (CUCRID), and hereby recommended for further processing.
Thank you.

For ______________________ ______________________
Chair, Ethical Committee **Member, Ethical Committee**
