# Supplementary material for: Data on information sources, knowledge and practice on hepatitis B virus in southwest Nigeria
Source: Data Brief. 2020 Apr 9;30:105507. doi: 10.1016/j.dib.2020.105507 (PMC7168725; doi:10.1016/j.dib.2020.105507)
Supplement: Supplementary file 4 [file mmc4.docx]

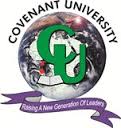


**Covenant University,**

**Department of Mass Communication,**

**Canaanland, Ota, Ogun State.**

**Dear Respondent,**

**This survey aims to explore respondents’ knowledge, attitude and practice towards Hepatitis B infection. Please, be assured that your responses will be treated as strictly confidential and for the purpose of research.**

**1. Have you heard about hepatitis B?**

1. **Yes**
2. **No**

**2. Do you seek information on hepatitis B from any mass media channels?**

1. **Yes**
2. **No**

**3. If yes, how frequently do you seek information about hepatitis B from the mass media channels?**

1. **Very often**
2. **Often**
3. **Rarely**
4. **Never.**

**4. What is the predominant channel you heard about hepatitis B**

1. **Television**
2. **Radio**
3. **Newspaper**
4. **Internet/ website**
5. **Friends**
6. **Colleagues**
7. **Relative**
8. **Spouse**
9. **Neighbors**
10. **Counselor**
11. **Health worker**
12. **Others, please specify________________________**

**5. Do you know what Hepatitis B means?**

1. **Yes**
2. **No**

**6. Hepatitis B is a/an**

1. **Eye disease**
2. **Brain disease**
3. **Liver disease**
4. **Bone disease**
5. **Skin disease**

**Kindly indicate your personal knowledge on the following questions. SD=Strongly Disagree, D=Disagree, U= Undecided, A=Agree, SA= Strongly Agree**

|  |  | **SD** | **D** | **U** | **A** | **SA** |
| --- | --- | --- | --- | --- | --- | --- |
| **7** | **Radio has provided me information on hepatitis B** |  |  |  |  |  |
| **8** | **Television programmes have provided me information on hepatitis B** |  |  |  |  |  |
| **9** | **Newspaper has provided me information on Hepatitis B** |  |  |  |  |  |
| **10** | **Leaflets/pamphlet /brochure/flyer/catalogue has/have provided me information on hepatitis B** |  |  |  |  |  |
| **11** | **Internet/ website has provided me information on hepatitis B** |  |  |  |  |  |
| **12** | **Health worker(s) has provided me information on hepatitis B** |  |  |  |  |  |
| **13** | **Colleague has given me information about hepatitis B** |  |  |  |  |  |
| **14** | **Neighbor(s) has/have provided me information about hepatitis B** |  |  |  |  |  |
| **15** | **Counselor has provided me information about hepatitis B** |  |  |  |  |  |
| **15** | **Relative has provided me information about hepatitis B** |  |  |  |  |  |
| **17** | **Seminar/workshop/conference organized has provided me information about hepatitis B** |  |  |  |  |  |

**Kindly indicate your personal knowledge on the following questions. SD=Strongly Disagree, D=Disagree, U= Undecided, A=Agree, SA= Strongly Agree**

|  |  | **SD** | **D** | **U** | **A** | **SA** |
| --- | --- | --- | --- | --- | --- | --- |
| **18** | **Hepatitis B is caused by a virus** |  |  |  |  |  |
| **19** | **Hepatitis B affect proper functioning of the liver** |  |  |  |  |  |
| **20** | **Hepatitis B can be transmitted from mother to foetus** |  |  |  |  |  |
| **21** | **Hepatitis can be transmitted through the use of infected sharp objects** |  |  |  |  |  |
| **22** | **Hepatitis B cause liver cancer** |  |  |  |  |  |
| **23** | **Jaundice is one of the common symptoms of Hepatitis B** |  |  |  |  |  |
| **24** | **Hepatitis B can be transmitted by contaminated water/food prepared by person suffering with these infections** |  |  |  |  |  |
| **25** | **A person can be infected with HIV and Hepatitis B at the same time** |  |  |  |  |  |
| **26** | **A person can be infected with Hepatitis B and not have the physical symptoms of the disease** |  |  |  |  |  |
| **27** | **Hepatitis B can be spread by having sexual intercourse with an infected person** |  |  |  |  |  |
| **28** | **There is a vaccine for Hepatitis** |  |  |  |  |  |
| **29** | **Hepatitis B can be cured** |  |  |  |  |  |

**(Attitude Level)**

|  |  | **SD** | **D** | **U** | **A** | **SA** |
| --- | --- | --- | --- | --- | --- | --- |
| **30** | **I have no concern being infected with hepatitis B** |  |  |  |  |  |
| **31** | **I think I can be infected with hepatitis B** |  |  |  |  |  |
| **32** | **I would be willing to go for hepatitis B test** |  |  |  |  |  |
| **32** | **I need to be protected from hepatitis B** |  |  |  |  |  |
| **33** | **I would be afraid if I find out I had hepatitis** |  |  |  |  |  |
| **34** | **Hepatitis B patients should be isolated** |  |  |  |  |  |
| **35** | **I engage in life style practices to prevent hepatitis B** |  |  |  |  |  |
| **36** | **I conduct preliminary health practices to prevent hepatitis B** |  |  |  |  |  |
| **37** | **If I think I have I have symptoms of hepatitis B I opt for self-medication** |  |  |  |  |  |
| **38** | **If I think I have I have symptoms of hepatitis B I will conduct study on hepatitis B** |  |  |  |  |  |
| **39** | **If I think I have I have symptoms of hepatitis B I will go to a health facility** |  |  |  |  |  |
| **40** | **If I think I have I have symptoms of hepatitis B I will go to a traditional healer** |  |  |  |  |  |
| **41** | **If I think I have I have symptoms of hepatitis B I go for spiritual healing** |  |  |  |  |  |
| **42** | **If I think I have I have symptoms of hepatitis B I will do nothing** |  |  |  |  |  |
| **43** | **If I think I have I have symptoms of hepatitis B, I will seek medical attention when I can no longer bear the thought of it** |  |  |  |  |  |
| **44** | **If I think I have I have symptoms of hepatitis B, I will seek medical attention When I run out of options on herbal cure** |  |  |  |  |  |
| **45** | **If I think I have I have symptoms of hepatitis B, I will seek medical attention When I find out how I contacted it** |  |  |  |  |  |
| **46** | **Fear of death would be my main concern if I am diagnosed with Hepatitis B** |  |  |  |  |  |
| **47** | **Fear of disease spreading to family members would be my main concern if I am diagnosed with Hepatitis B** |  |  |  |  |  |
| **48** | **Cost of treatment would be my main concern if I am diagnosed with Hepatitis B** |  |  |  |  |  |
| **49** | **The stigma and isolation from social life would be my main concern if I am diagnosed with Hepatitis B** |  |  |  |  |  |

**(Practice Level)**

**Kindly indicate your personal knowledge on the following questions. SD=Strongly Disagree, D=Disagree, U= Undecided, A=Agree, SA= Strongly Agree**

|  |  | **SD** | **D** | **U** | **A** | **SA** |
| --- | --- | --- | --- | --- | --- | --- |
| **50** | **I have been screened for hepatitis B before** |  |  |  |  |  |
| **51** | **I have been vaccinated against hepatitis B** |  |  |  |  |  |
| **52** | **I ask for a new syringe at health facilities if you need to use one** |  |  |  |  |  |
| **53** | **I often ask my hair stylist to change blade, needle or sterilize the clipper** |  |  |  |  |  |
| **54** | **I often insist on the use of sterilized or safe objects for any form of body piercing (e.g. ear, nose)** |  |  |  |  |  |
| **55** | **I ask about screening of blood before transfusion** |  |  |  |  |  |
| **56** | **If I am diagnosed with Hepatitis B, I would you proceed on further medical consultations** |  |  |  |  |  |
| **57** | **If I am diagnosed with Hepatitis B, I would share food utensils with someone else** |  |  |  |  |  |
| **58** | **If I am diagnosed with Hepatitis B, I would avoid meeting with people** |  |  |  |  |  |

**59. Have you ever participated in a health education program/ campaign related to Hepatitis B?**

1. **Yes**
2. **No**
3. **Not sure**

**Demographics**

**60. Age**

1. **Below 18**
2. **18-25**
3. **26-35**
4. **36-45**
5. **46-55**
6. **55- Above**

**61. Sex**

1. **Male**
2. **Female**

**62. Highest educational qualification**

1. **NCE**
2. **Ordinary National Diploma**
3. **Higher National Diploma**
4. **B.Sc, B.Ed, B.Tech**
5. **M.A, M.Sc, M.Ed, M.Phil, Ph.D**

**63. Occupation**

1. **Trader**
2. **Artisan**
3. **White collar job**
4. **Student**
5. **Unemployed**
6. **Others**

**64. Religion**

1. **Christianity**
2. **Islam**
3. **Traditional**
4. **Others ________________**

**65. Marital Status**

1. **Single**
2. **Married**
3. **Single parent**
4. **Others ________________**
